# Dual energy X-ray beam ptycho-fluorescence imaging

**DOI:** 10.1107/S1600577521008675

**Published:** 2021-10-05

**Authors:** Silvia Cipiccia, Francesco Brun, Vittorio Di Trapani, Christoph Rau, Darren J. Batey

**Affiliations:** a Department of Medical Physics and Biomedical Engineering, University College London, Gower Street, London WC1E 6BT, United Kingdom; b Diamond Light Source, Harwell Science and Innovation Campus, Fermi Avenue, Didcot OX11 0QX, United Kingdom; c Department of Engineering and Architecture, University of Trieste, Via Alfonso Valerio 6/1, Trieste 34127, Italy; d Department of Physics, University of Trieste, Via Alfonso Valerio 6/1, Trieste 34127, Italy

**Keywords:** X-ray fluorescence, X-ray ptychography, pink beam

## Abstract

X-ray ptychography and X-ray fluorescence are complementary nanoscale imaging techniques, providing structural and elemental information, respectively. Both methods acquire data by scanning a localized beam across the sample. X-ray ptychography processes the transmission signal of a coherent illumination interacting with the sample, to produce images with a resolution finer than the illumination spot and step size. By enlarging both the spot and the step size, the technique can cover extended regions efficiently. X-ray fluorescence records the emitted spectra as the sample is scanned through the localized beam and its spatial resolution is limited by the spot and step size. The requisites for fast ptychography and high-resolution fluorescence appear incompatible. Here, a novel scheme that mitigates the difference in requirements is proposed. The method makes use of two probes of different sizes at the sample, generated by using two different energies for the probes and chromatic focusing optics. The different probe sizes allow to reduce the number of acquisition steps for the joint fluorescence–ptychography scan compared with a standard single beam scan, while imaging the same field of view. The new method is demonstrated experimentally using two undulator harmonics, a Fresnel zone plate and an energy discriminating photon counting detector.

## Abstract 2

A novel scheme for simultaneous X-ray ptychographic and fluorescence imaging is presented. The method is based on a dual energy X-ray beam, chromatic focusing optics and an energy discriminating photon counting detector.

## 1. Introduction

X-ray ptychography (Rodenburg *et al.*, 2007 ▸) and X-ray fluorescence (XRF) imaging techniques have been successfully combined to produce simultaneously high resolution structural images and elemental distribution maps in 2D (Vine *et al.*, 2012 ▸; Deng *et al.*, 2015 ▸, 2017 ▸) and 3D (Deng *et al.*, 2018 ▸; Victor *et al.*, 2018 ▸). Despite both being scanning techniques, the scanning requirements are different. In X-ray ptychography, the sample is scanned with respect to the beam in an overlapping fashion while recording the diffraction pattern intensity in the far field. The overlap generates an information-rich dataset that allows for robust solutions to the phase problem through iterative algorithms (Marchesini *et al.*, 2016 ▸; Enders & Thibault, 2016 ▸; Maiden & Rodenburg, 2009 ▸; Guizar-Sicairos & Fienup, 2008 ▸). A typical beam overlap in a ptychographic scan ranges between 60 and 80% (Edo *et al.*, 2013 ▸). The illumination size at the sample and the achievable resolution depend on both the X-ray energy and the experimental geometry. In coherent diffraction imaging (CDI), the maximum spatial extent of the illumination at the sample (modulus), *D*, is limited by the Nyquist sampling condition. The sampling pitch in the far field must be fine enough to resolve the interference fringes from the edges of the illuminating beam. Therefore, *D* is given by

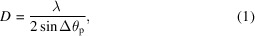

where λ is the X-ray wavelength, and Δθ_p_ the angle subtended by the detector pixel-pitch. Ptychography can deal with larger illuminations than CDI, by tuning the scanning step size (Edo *et al.*, 2013 ▸; Batey, 2014 ▸; da Silva & Menzel, 2015 ▸). However, when an illumination larger than that specified by equation (1) is used, the reconstruction algorithm requires up-sampling (Batey *et al.*, 2014 ▸) and becomes more computationally expensive. Therefore, the CDI sampling condition is generally used experimentally for setting up a ptychographic scan, and it was used also in the experiment presented here.

The reconstructed pixel size *d* depends on the wavelength λ and the angle subtended by the detector active area, α,

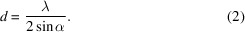

Conversely, in XRF imaging, the pixel size is defined by the scanning step size, the resolution depends on the size of the beam intensity profile at the sample, and the technique does not require beam overlap. XRF images with nanoscale resolution have been produced around the world at nanoprobe beamlines (Chen *et al.*, 2016 ▸; Victor *et al.*, 2018 ▸; Steinmann *et al.*, 2020 ▸; Rumancev *et al.*, 2020 ▸). When combining XRF and X-ray ptychography, the requirements for the beam size versus step size ratio do not match. Ptychography requires a 2.5–5:1 ratio (based on an overlap of 60–80%), while XRF requires a 1:1 ratio. When using a beam larger than the step, the XRF resolution is no longer step limited but dominated by the beam size. Knowledge of the probe, provided by ptychography, can be used to deconvolute the fluorescence image and improve the resolution (Vine *et al.*, 2012 ▸), mitigating the different scanning requirements.

We propose a new method that allows further improvement of the simultaneous XRF-ptycho scanning parameters, by reducing the number of scanning steps and the data acquired. The method is based on the following specifications:

(i) The X-ray beam spectrum consists of two energy peaks, *E*_1_ and *E*_2_, with *E*_1_ < *E*_2_, separated by *ΔE*; *E*_1_ is used for ptychography and *E*_2_ to excite the XRF.

(ii) *E*_1_ and *E*_2_ have a different beam size at the sample, *D*_1_ and *D*_2_, respectively. *D*_1_ satisfies equation (1), while *D*_2_ is equal to the ptychography scanning step size (20% of *D*_1_ for 80% overlap).

(iii) An energy resolving X-ray photon counting detector (XPCD) with energy resolution better than Δ*E* records in the far field the diffraction pattern for *E*_1_ while removing *E*_2_.

We have carried out an experimental proof of the proposed configuration at the I13-1 beamline of Diamond Light Source by imaging a gold Siemens star resolution target, using the Pixirad-1/Pixie-III XPCD (Bellazzini *et al.*, 2015 ▸).

## 2. Method

The I13-1 beamline is specialized in multimodal 3D ptychography (Cipiccia *et al.*, 2019 ▸; Martin *et al.*, 2019 ▸; Weber *et al.*, 2020 ▸). The X-ray source consists of a 2.8 m long, 25 mm period undulator. The mini-beta configuration (Rau, 2017 ▸) combined with the large source-to-sample distance of 215 m provides a high level of coherent flux. The transverse coherence length in the experimental hutch is tunable by adjusting a set of front-end slits. The beamline is equipped with a triple-stripe mirror (Si, Pt, Ro), a metal filter box and a Si(111) double-crystal monochromator (DCM). The beamline can be operated either in monochromatic or pink beam mode by removing the DCM from the X-ray path (Batey *et al.*, 2019 ▸; Brun, Di Trapani, Batey *et al.*, 2020 ▸).

### 2.1. Pixirad detector

Pixirad-1/Pixie-III is a direct detection XPCD built with a hybrid architecture, where a semiconductor sensor is coupled with the flip-chip bonding technique to the ASIC. The 650 µm thick CdTe sensor is a Schottky type diode array with electron collection on the pixels. The detector active area (3.17 cm × 2.49 cm) consists of a 512 × 402 matrix of square pixels with 62 µm pitch. Pixirad-1/Pixie-III implements a specifically designed acquisition mode that improves both the energy resolution and the detection efficiency by compensating for the charge sharing issue (Delogu *et al.*, 2016 ▸; Di Trapani *et al.*, 2018 ▸, 2020 ▸). By implementing two programmable energy thresholds (thr_1_ and thr_2_) the detector can operate in one- or two-colours mode. In one-colour mode, a single threshold is set: all the events with energy above this global threshold are collected in a single image. In two-colours mode, Pixirad outputs two images in a single acquisition: one collects events with energy between thr_1_ and thr_2_, the second collects events with energy above thr_2_. The detector has proved its effectiveness for single-shot *K*-edge subtraction computed tomography (KES-CT) with polychromatic laboratory sources (Brun, Di Trapani, Albers *et al.*, 2020 ▸) and has been recently used as a harmonic selector for edge subtraction ptychographic imaging using synchrotron X-ray pink beam (Brun, Di Trapani, Batey *et al.*, 2020 ▸).

### 2.2. Experimental setup

**Figure 1:**
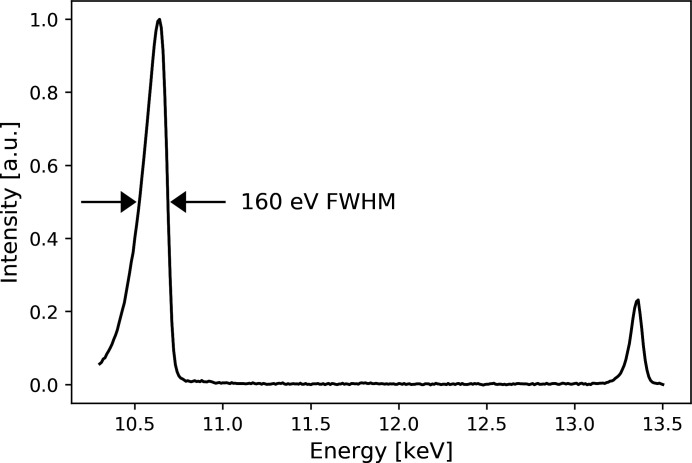
Filtered undulator spectrum measured in the experimental hutch showing the two energies *E*_1_ and *E*_2_. The X-ray intensity was measured with an ionization chamber placed before the sample while scanning the X-ray energy with the DCM.

We conducted the experiment using pink beam. The undulator gap was set to 13.0 mm which produces the fourth harmonic at 10.64 keV (*E*_1_) and fifth harmonic at 13.35 keV (*E*_2_) (see spectrum in Fig. 1 ▸), with Δ*E* = *E*_2_ − *E*_1_ larger than the energy resolution of Pixirad [2.3 keV energy spread measured at 11 keV (Di Trapani *et al.*, 2020 ▸)]. All the undulator harmonics but *E*_1_ and *E*_2_ were suppressed using a combination of filters and mirror: 1.34 mm of pyrolytic graphite, 0.7 mm of aluminium and the Si mirror strip. *E*_2_, which is above the *L*_III_ absorption edge of gold (11.92 keV), is used for the XRF to excite the fluorescence from the test sample.

**Figure 2:**
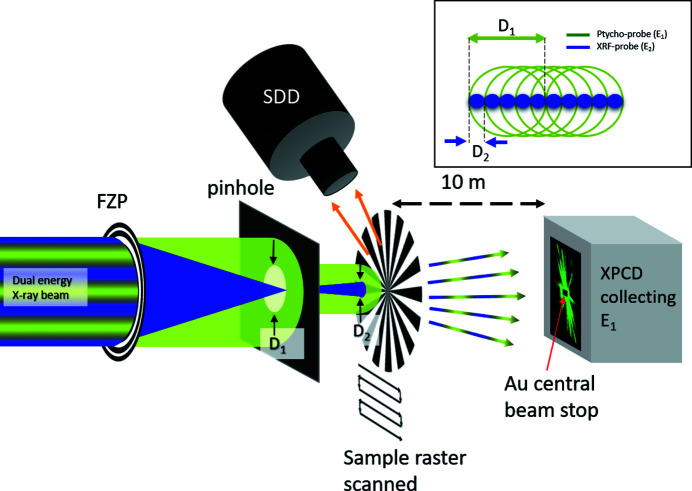
Setup, not to scale, for the dual beam ptycho-fluorescence imaging method. The two energies *E*_1_ (green) and *E*_2_ (blue) have a different beam size at the sample, *D*_1_ and *D*_2_, respectively. The XPCD detector in the far field records the diffraction patterns for *E*_1_, while the central beam stop blocks the direct beam. Inset: schematic of the beams overlap in the dual beam scan method, where the probe size for the XRF (blue) matches the step size of the ptychography scan (green).

The experimental setup is illustrated in Fig. 2 ▸, where *E*_1_ is shown in green and *E*_2_ in blue. High efficiency (∼20%) blazed gold Fresnel zone plates (FZPs) (Gorelick *et al.*, 2011 ▸) with 400 µm diameter, 150 nm outermost width, were used to focus *E*_2_. In I13-1 the X-ray beam is astigmatic because of the mini-beta configuration (Rau, 2017 ▸), and in order to produce a symmetric beam for the experiment we operated out-of-focus for the XRF, with a spot size at the sample of *D*_2_ = 2 µm. A 10 µm pinhole placed at the FZP focal position for *E*_2_ blocks the diffraction orders >1 for *E*_2_. At the same time the pinhole defines the illumination spot *D*_1_ for *E*_1_, by letting pass only its zero order. *D*_1_ = 10 µm satisfies the sampling requirements of equation (1) and *D*_2_ = 2 µm matches the ptychography scanning step for 80% overlap of *D*_1_ (see inset in Fig. 2 ▸).

The gold Siemens star test sample mounted on a six-axis sample stage was scanned at 2 µm steps in a 38 × 38 raster grid at 0.9 Hz. The fluorescence excited by *E*_2_ was collected with a single-element silicon drift Vortex detector placed at a slight angle from the sample plane, to minimize the self-absorption. A 10 m helium-filled pipe was placed between the sample and Pixirad detector, to reduce the air scattering of the diffracted beam. A 700 µm diameter, 100 µm thick gold central beam stop was positioned in front of the detector to block the direct beam. The two thresholds of Pixirad were set, respectively, to 9 keV and 10 keV to select the diffraction pattern of *E*_1_ in the far field. thr_2_ was set below *E*_1_ to minimize the contamination from *E*_2_ due to the finite energy resolution of the XPCD, at the cost of a loss in efficiency for the peak *E*_1_.

**Figure 3:**
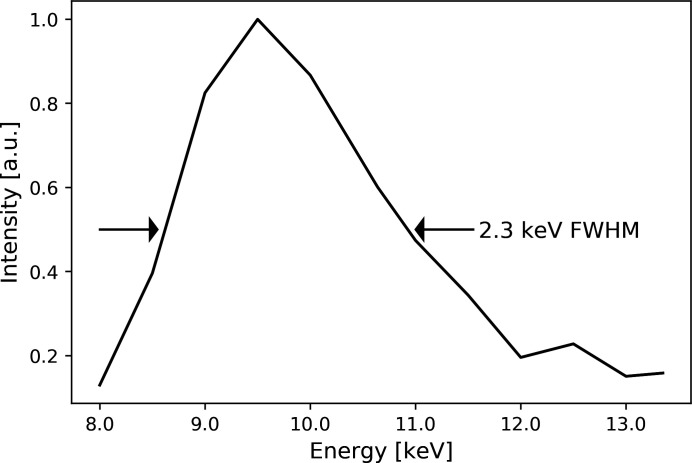
Measured detector effective bandwidth: FWHM 2.3 keV. The integrated number of counts is normalized by the beam intensity measured upstream of the sample and optics with an ionization chamber.

The effective energy bandwidth of the detector under the applied thresholds was measured by scanning the X-ray beam energy with the DCM between 8.5 and 13.5 keV. The bandwidth of the detector was measured to be 2.3 keV FWHM, as shown in Fig. 3 ▸, in good agreement with the literature (Di Trapani *et al.*, 2020 ▸).

## 3. Results

**Figure 4:**
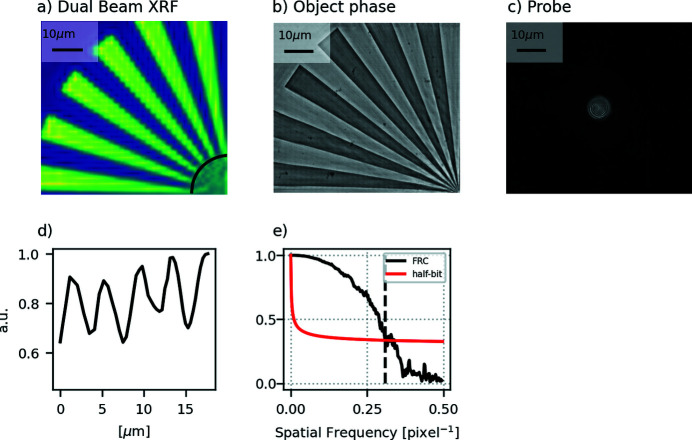
Ptycho-fluorescence results: (*a*) Fluorescence image of the Siemens star obtained with dual beam setups. (*b*–*c*) Ptychographic reconstruction of the phase image of the Siemens star and probe modulus obtained with the energy filtered data. Reconstructed pixel size: 77 nm. (*d*) Line profile for the fluorescence image showing the resolution of 4 µm limited by the smallest beam size *D*_2_ = 2 µm. (*e*) Fourier ring correlation analysis for the ptychography reconstruction showing a resolution of 0.31 pixel^−1^, corresponding to 248 nm.

The results of the scan performed with the dual beam method are shown in Fig. 4 ▸. A 38 × 38 pixel fluorescence image with a pixel size of 2 µm [Fig. 4 ▸(*a*)] was produced from the spectrum acquired by the Vortex, using an in-house script. The spatial resolution for the fluorescence image is 4 µm, as can be seen by the line profile of Fig. 4 ▸(*d*). The ptychography reconstruction for the energy filtered data from a 256 × 256 pixel region of the detector was performed with 1000 iterations of ePIE through the PtyREX package (Batey, 2014 ▸). The output object phase and the modulus of the illumination are shown in Figs. 4 ▸(*b*) and 4 ▸(*c*): the reconstructed pixel size is 77 nm and a resolution of 248 nm was estimated with Fourier ring correlation as shown in Fig. 4 ▸(*e*).

The results show that the dual beam method succeeded in generating different probe size at the sample: for XRF, *D*_2_ = 2 µm allows for a resolution of 4 µm while, for ptychography, the illumination size is retrieved by the reconstruction algorithm from the detector filtered pink beam data to be *D*_1_ = 10 µm.

## 4. Discussion

We have presented here a new method for simultaneous X-ray ptychography and XRF imaging, that mitigates the difference in scanning requirements for the two techniques, using a dual energy beam with different energies and illumination sizes at the sample. Once the resolution for the XRF is chosen (that is the smallest beam size), the second beam can be set five times larger to perform ptycho-XRF simultaneously with a reduced number of data points and acquisition time.

We have verified experimentally the method by generating a dual beam, and using an energy discriminating detector for separating the energies in the far field. A standard single beam setup is expected to require up to 25 times more acquisitions, reducing possible sources of overhead and therefore scanning time and data to perform combined XRF and ptychography, to image the same field of view.

The dual beam was generated using a FZP and pinhole combination: in this setup no beam stop can be used before the sample, which otherwise would block *E*_1_. Consequently, also the zero order of *E*_2_ reaches the sample and it could blur the XRF resolution. However, the FZPs used in the experiment are optimized for the first diffraction order and the intensity of the zero order is negligible compared with the first (Gorelick *et al.*, 2011 ▸).

The experiment was performed using pink beam and the bandwidth of the fourth harmonic used for the ptychographic imaging was 160 eV (Fig. 1 ▸), which corresponds to a maximum achievable resolution of 150 nm (Spence *et al.*, 2004 ▸). In the reported experiment, the spatial resolution for the ptychography imaging was measured to be 248 nm and therefore not limited by the bandwidth. The resolution limiting factor was the photon statistic: using a pinhole for defining the ptychography illumination did not allow to make full use of the available coherent flux. To counterbalance the low photon statistic, we set the exposure time to 1.1 s and, because of the low acquisition rate (0.9 Hz), a step scan was performed instead of a fly scan. However, the method itself is independent of the type of scan, step or fly. Since there was no beam stop before the sample, a beam stop in front of the detector was required to block the direct beam from damaging the sensor. The beam stop at the detector removes the low spatial frequencies of the ptychographic dataset which generates a halo around the larger spokes of the Siemens star in the reconstructed object phase, as observable in Fig. 4 ▸(*b*).

For the first proof of principle, the FZP and pinhole configuration was preferred because it is easy to resource and to implement. The setup was out-of-focus for the XRF; this choice was driven by the specific feature of the I13-1 instrument (astigmatic beam), however the method can be applied to in-focus configurations.

The system could be improved by replacing the FZP and pinhole configuration with a system of 2 × 1/2 off-axis FZPs to generate the different probe size at the sample for the two energies (Döring *et al.*, 2019 ▸). A 1/2 FZP and 1/2 FZP configuration would allow using a beam stop before the sample instead of at the detector, solving the issue of the missing low spatial frequencies. Moreover, a 1/2 FZP and 1/2 FZP configuration would allow to make a better use of the coherent flux by matching the lateral coherence length with the optics aperture. The higher flux would allow shorter exposure suitable for faster fly scanning acquisition, enable higher resolution, and finally would provide extra flexibility in defining the ptychography beam size, to suit also in-focus XRF scans, particularly relevant at nanoprobe beamlines.
